# A Double Extended Kalman Filter Algorithm for Weakening Non-Line-of-Sight Errors in Complex Indoor Environments Based on Ultra-Wideband Technology

**DOI:** 10.3390/s25030740

**Published:** 2025-01-26

**Authors:** Sheng Xu, Qianyun Liu, Min Lin, Qing Wang, Kaile Chen

**Affiliations:** 1 School of Instrument Science and Engineering, Southeast University, Nanjing 210096, China; xus@jitri.cn (S.X.); wq_seu@seu.edu.cn (Q.W.); 2 Advanced SoC and IoT Technology Laboratory (ASITLAB), Shanghai University, Shanghai 200444, China; lqy1239079631@163.com (Q.L.); ckl1108@shu.edu.cn (K.C.); 3 Key Laboratory of Specialty Fiber Optics and Optical Access Networks, Shanghai University, Shanghai 200444, China

**Keywords:** ultra-wideband (UWB), non-line-of-sight (NLOS), time of arrival (TOA), Kalman filter (KF), residual classification, covariance adjustment

## Abstract

In complex indoor environments, target tracking performance is impacted by non-line-of sight (NLOS) noises and other measurement errors. In order to fix NLOS errors, a double extended Kalman filter (DEKF) algorithm is proposed, which refers to a kind of cascaded structure composed of two Kalman filters. In the proposed algorithm, the first filter is a classic Kalman filter (KF) and the second is an extended Kalman filter (EKF). Time of arrival (TOA) measurements collected by multiple stationary ultra-wideband (UWB) sensors are used. The residual errors between the measured TOA and that of the first KF are predicted, and the covariance of the first KF is adjusted correspondingly. Then, we use the estimated distance state of the first KF as a measurement vector for the second EKF in order to obtain a smoother observation. One of the advantages of the proposed algorithm is that it is able to perform target tracking with good accuracy even without or with only one LOS TOA measurement for a period of time without prior information about the NLOS noise, which may be difficult to obtain in practical applications. Another advantage is that the accuracy does not greatly decrease when NLOS noises exist for a long period of time. Finally, the proposed DEKF can maintain the high-precision positioning characteristics in both the constant velocity (CV) model and the constant acceleration (CA) model in the LOS/NLOS environment. Our simulation and experimental results show that the proposed algorithm performs much better than other algorithms in SOTA, particularly in severe mixed LOS/NLOS environments.

## 1. Introduction

With the development of wireless communication technology, the demand for location-based services (LBSs) is rapidly increasing [1,2,3,4,5]. Currently, there are several technologies for indoor positioning, such as Bluetooth Low Energy (BLE) [6,7,8], WiFi [9,10,11], Radio Frequency Identification (RFID) [12,13,14], and Impulse Radio–Ultra Wideband (IR-UWB) [15,16,17,18]; IR-UWB is superior to the others with its centimeter positioning accuracy in complex indoor environments.

The UWB signals are impacted by multipath effects, which may lead to great measurement errors, especially in NLOS environments. The algorithm proposed in [19] keeps the LOS pseudo-measurement results, discarding the NLOS ones, and calculates the average of all the selected LOS measurements. The limitation of this tracking algorithm is that, when there is no LOS BaseStation within a period of time, the estimation of states can only rely on predictions. The Kalman filter and its enhancements, such as EKF, cubature Kalman filter (CKF), unscented Kalman filter (UKF), adaptive Kalman filter (AKF), or particle filter (PF), are used [20,21,22,23,24,25,26,27] to reduce NLOS noise-introduced errors in complex indoor environments [28,29]. To reduce measurement errors, forms of extended KF (EKF) with robust estimation, such as M-estimation and fuzzy-tuning M-estimation EKF, were proposed in [30]. However, the above filtering technologies are still limited by their accuracy, especially in complex LOS and NLOS mixture conditions. A time-of-flight (ToF)-based NLOS indoor tracking method incorporating adaptive ranging error mitigation techniques is presented in [31]. However, due to its high computational complexity, it is difficult to appropriately apply this method to scenarios with high real-time requirements. The classification of LOS and NLOS conditions using deep learning (DL) models was mentioned in [32]. However, the study does not discuss the performance of the model in complex indoor environments in detail. Deep-learning-based NLOS/LOS classification methods for indoor positioning systems were also mentioned in [33]. However, the training of deep learning models requires a large amount of data support, and it may be a challenge to obtain enough labeled data in some indoor environments.

In order to further improve target tracking performance in complex LOS and NLOS mixture conditions, a double Kalman filter with a residual classification and covariance adjustment (RCCA) algorithm is proposed to mitigate NLOS-introduced errors. In this algorithm, two Kalman filters are cascaded, where the first filter is a KF and the second is an EKF. The RCCA process is applied on the KF system, whose states are distances and velocities. Residual classification means that the residuals between the first KF’s observed measurements and its system predictions are calculated, and then, the covariance of the first KF system is adjusted for the Kalman gain according to the error ranges. After that, the distance vectors are used as measurements of the second EKF, and the estimated variance of the first KF is input as the measurement covariance. This completes the RCCA process.

The remainder of this article is organized as follows. In Section 2, we introduce the system framework of this methodology. In Section 3, we discuss the residual classification and variance adjustment process. Section 4 presents the details of the DEKF algorithm. In Section 5, the simulation results are shown and experimental verification is carried out. Finally, Section 6 presents the conclusions.

## 2. System Model

### 2.1. Extended Kalman Filter Modeling

A system containing one target and M stationary UWB anchors is considered in this section. For generality, the constant acceleration (CA) model is used, so the state of target can be defined as follows:

(1) $$X_{k}={\left[x_{k},y_{k},z_{k},{\dot{x}}_{k},{\dot{y}}_{k},{\dot{z}}_{k},{\ddot{x}}_{k},{\ddot{y}}_{k},{\ddot{z}}_{k}\right]}^{T},$$

where ${\left[x_{k},y_{k},z_{k}\right]}^{T}$ is the coordinate of the target, ${\left[{\dot{x}}_{k},{\dot{y}}_{k},{\dot{z}}_{k}\right]}^{T}$ is the velocity of the target, ${\left[{\ddot{x}}_{k},{\ddot{y}}_{k},{\ddot{z}}_{k}\right]}^{T}$ is the acceleration of the target, and k stands for the present moment. The stationary UWB anchors are located at $X_{i}^{U}={\left[x_{i}^{U},y_{i}^{U},z_{i}^{U}\right]}^{T},i=1,\ldots ,M$. In order to simplify the analysis, a constant velocity (CV) model is used for formula derivation, which is essentially equivalent to the CA model with acceleration equal to zero. Thus, the state of the target is defined as follows:

(2) $$X_{k}={\left[x_{k},y_{k},z_{k},{\dot{x}}_{k},{\dot{y}}_{k},{\dot{z}}_{k}\right]}^{T},$$

The measured distances between the target and the anchors are calculated using the received time of flight (TOF), which is expressed as $Z_{k}=c\cdot {\mathrm{TOF}}_{k}$, $c=3\times 10^{8}$ (m/s). The state evolution model of the EKF system is as follows:

(3) $$X_{k}=AX_{k-1}+w_{k},$$

(4) $$Z_{k}=h\left(X_{k}\right)+v_{k},$$

where A is the transition matrix of the states in the CV model:

(5) $$A=\left[\begin{array}{cccccc} 1 & 0 & 0 & T_{s} & 0 & 0\\ 0 & 1 & 0 & 0 & T_{s} & 0\\ 0 & 0 & 1 & 0 & 0 & T_{s}\\ 0 & 0 & 0 & 1 & 0 & 0\\ 0 & 0 & 0 & 0 & 1 & 0\\ 0 & 0 & 0 & 0 & 0 & 1 \end{array}\right],$$

where $T_{s}$ is the observation time of the system and $h\left(X_{k}\right)={\left[h_{1}\left(X_{k}\right),\ldots ,h_{M}\left(X_{k}\right)\right]}^{T}$ is a non-linear transfer function of state and measurement, where $h_{i}\left(X_{k}\right)$ = $\sqrt{{\left(x_{k}-x_{i}^{U}\right)}^{2}+{\left(y_{k}-y_{i}^{U}\right)}^{2}+{\left(z_{k}-z_{i}^{U}\right)}^{2}}$. The essence of EKF is to transform the non-linear case into a locally linear case. For non-linear functions, the properties of derivatives can be exploited for local linearization by a Jacobian matrix. The Jacobian can be obtained by taking the partial derivative with respect to each dimension of $h_{i}\left(X_{k}\right)$. It is shown below:

(6) $$H_{k}=\left[\begin{array}{cccccc} \frac{x_{k}-x_{1}^{U}}{h_{1}\left(X_{k}\right)} & \frac{y_{k}-y_{1}^{U}}{h_{1}\left(X_{k}\right)} & \frac{z_{k}-z_{1}^{U}}{h_{1}\left(X_{k}\right)} & 0 & 0 & 0\\ \vdots & \vdots & \vdots & \vdots & \vdots & \vdots \\ \frac{x_{k}-x_{M}^{U}}{h_{M}\left(X_{k}\right)} & \frac{y_{k}-y_{M}^{U}}{h_{M}\left(X_{k}\right)} & \frac{z_{k}-z_{M}^{U}}{h_{M}\left(X_{k}\right)} & 0 & 0 & 0 \end{array}\right]$$

The non-linear function $h_{i}\left(X_{k}\right)$ does not contain ${\left[{\dot{x}}_{k},{\dot{y}}_{k},{\dot{z}}_{k}\right]}^{T}$, and thus, the right-hand side of the matrix is an M × 3 zero matrix.

In (3), $w_{k}$ is a process noise vector with a covariance matrix $Q_{k}^{x}$. In (4), $v_{k}$ is a measurement noise vector with a covariance matrix $R_{k}^{x}$, which is determined by the measurement accuracy of the anchors. As for the value of $Q^{x}$, which is mentioned in [34], it takes different values in different models. In the CV model, it is defined as follows according to [34]:

(7) $$Q^{x}=\Phi_{s1}\left[\begin{array}{cc} \frac{T_{s}^{3}}{3}I_{3} & \frac{T_{s}^{2}}{2}I_{3}\\ \frac{T_{s}^{2}}{2}I_{3} & T_{s}I_{3} \end{array}\right].$$

where $\Phi_{s1}$ is the spectrum density of the process noise for the KF system and $I_{3}$ is a 3 × 3 unit matrix.

The iterative computation process for the EKF system is as follows [20,35,36]:

(8) $$\begin{array}{c} {\hat{X}}_{k|k-1}=A{\hat{X}}_{k-1}, \end{array}$$

(9) $$\begin{array}{c} {\hat{P}}_{k|k-1}^{x}=A{\hat{P}}_{k-1}^{x}A^{T}+Q_{k}^{x}, \end{array}$$

(10) $$\begin{array}{c} K_{k}^{x}={\hat{P}}_{k|k-1}^{x}H_{k}^{T}{\left(H_{k}{\hat{P}}_{k|k-1}^{x}H_{k}^{T}+R_{k}^{x}\right)}^{-1}, \end{array}$$

(11) $$\begin{array}{c} {\hat{X}}_{k}={\hat{X}}_{k|k-1}+K_{k}^{x}\left(Z_{k}-h\left({\hat{X}}_{k|k-1}\right)\right), \end{array}$$

(12) $$\begin{array}{c} {\hat{P}}_{k}^{x}=\left(I-K_{k}^{x}H_{k}\right){\hat{P}}_{k|k-1}^{x}. \end{array}$$

Equations (8) and (9) are update equations from the k−1 state to the *k* state, and (10), (11), and (12) are measurement update equations. ${\hat{P}}_{k-1}^{x}$ is the error covariance matrix of ${\hat{X}}_{k-1}$, A is the state transition matrix, $Q_{k}^{x}$ is the process noise covariance matrix, $R_{k}^{x}$ is the measurement noise covariance matrix, $H_{k}$ is the measurement transition matrix, $Z_{k}$ is the measurement matrix, and the weighting matrix $K_{k}^{x}$ is generally referred to as the Kalman gain matrix.

### 2.2. Measurement Error Modeling

**Figure 1:**
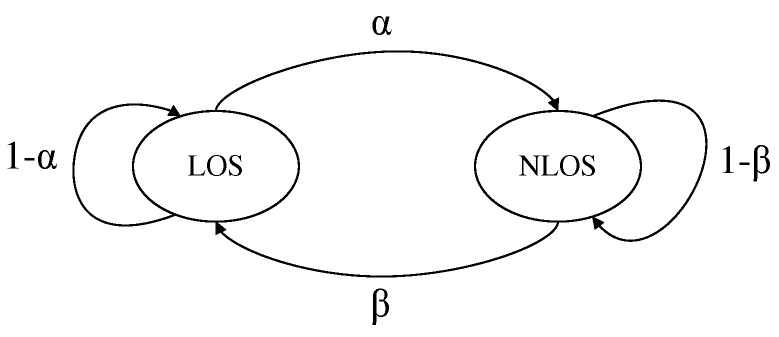
Markov process for LOS/NLOS transition.

Generally, measurement errors are made of measurement noise and NLOS noise, where measurement noise is modeled by Gaussian white noise determined by measured anchors. NLOS noise is caused by reflected, refracted, or multipath transmission signals. The measurement error modeling is as follows:

(13) $$\rho_{k,i}={\dot{\rho }}_{k,i}+n_{k,i}+w_{k,i},\;i=1,\cdots ,M.$$

where $\rho_{k,i}$ is the measured distance between the target and anchor *i*, ${\dot{\rho }}_{k,i}$ is the real distance, $n_{k,i}$ is the measurement noise, and $w_{k,i}$ is the NLOS noise. The NLOS noise is usually modeled with a uniform distribution, mean-shifted Gaussian distribution, or exponential distribution [19,30,36]. In the modeling, we assume a uniformly distributed NLOS noise in the following constraints for simplicity, although there are other methods for describing the NLOS noise, such as the Markov process. For a mixed LOS/NLOS environment, the probability density function of $\eta_{i}$ is defined as:

(14) $$p\left(\eta_{i}\right)=\left(1-\varepsilon_{i}\right)p_{\mathrm{LOS}}\left(n_{i}\right)+\varepsilon_{i}p_{\mathrm{NLOS}}\left(w_{i}\right),$$

where $\eta_{i}=n_{i}+w_{i}$ is defined as in (13), $\varepsilon_{i}$ is the proportion of NLOS errors at anchor *i*, $p_{\mathrm{LOS}}\left(n_{i}\right)$ is zero mean Gaussian distributed, and $p_{\mathrm{NLOS}}\left(w_{i}\right)$ is mean-shifted Gaussian distributed. The mixed LOS/NLOS model is implemented using a two-state Markov process, which is the same as that mentioned in [19,36,37,38]. The state transition is shown in Figure 1.

The transition probability matrix of the Markov process is:

(15) $$\left[\begin{array}{cc} 1-\alpha & \alpha \\ \beta & 1-\beta \end{array}\right],$$

where α is the probability of transition from state LOS to NLOS and β is the probability of transition from state NLOS to LOS. $\varepsilon_{i}$ is related to α and β by:

(16) $$\varepsilon_{i}=\frac{\alpha }{\alpha +\beta }.$$

The proportion of LOS and NLOS noise can be adjusted by the values of α and β, and there are several sets of values that satisfy one $\varepsilon_{i}$. Table 1 shows one possible combination.

## 3. Residual Classification and Covariance Adjustment

For the first Kalman filter of the two cascaded filters, the distances between the target and the anchors as well as their variation rates of measurement are state variables of KF:

(17) $$\begin{array}{c} Y_{k}={\left[\rho_{1,k},\cdots ,\rho_{M,k},{\dot{\rho }}_{1,k},\cdots ,{\dot{\rho }}_{M,k}\right]}^{T}. \end{array}$$

where $Y_{k}$ is the KF state vector in the *k*-th iteration, $\rho_{i,k}$ is the measured distance between the *i*-th anchor and the target in the *k*-th iteration, and ${\dot{\rho }}_{i,k}$ is the rate of change in the measured distance.

After calculating the prediction of the states, a residual obtained between measurement and prediction is shown below:

(18) $$\begin{array}{c} d_{k}=\left|Z_{k}-T{\hat{Y}}_{k|k-1}\right|, \end{array}$$

where $Z_{k}$ is a set of measurements collected by several stationary UWB anchors and T is a transition matrix between state and observation.

(19) $$T=\left[\begin{array}{cc} I_{M} & 0_{M} \end{array}\right].$$

Then, the range of the obtained residual is classified as follows:

$$\;d_{k}\{\begin{array}{cc} 0<max(d_{k})<\mu_{1} & \;(20a)\\ \mu_{1}\leq max(d_{k})<\mu_{2} & \;(20b)\\ \mu_{2}\leq max(d_{k})<\mu_{3} & \;(20c)\\ \vdots & \;(20d)\\ \mu_{N-3}\leq max(d_{k})<\mu_{N-2} & \;(20e)\\ \mu_{N-2}\leq max(d_{k})<\mu_{N-1} & \;(20f)\\ \mu_{N-1}\leq max(d_{k})<\mu_{N} & \;(20g) \end{array}$$

where $\mu_{N}>\mu_{N}-1>\cdots >\mu_{3}>\mu_{2}>\mu_{1}$ and *N* is the number of range groups. The classification criteria are based on the order of μ’s magnitude, and the choice of the first parameter $\mu_{1}$ in (20a) is based on the LOS measured noise covariance, which is determined by the UWB ranging accuracy. When the LOS measured noise is Gaussian distributed with (μ,σ)=(0m,0.1m), the cumulative distribution function (CDF) is defined as [39]:

(21) $$\begin{array}{c} F(a)=P(X\leq a). \end{array}$$

When a = 0.375, F(a) = 0.9999. This value is already very close to 1. When a is greater than 0.7, the sensitivity to noise is reduced. Therefore, 0.5 is selected as the value of $\mu_{1}$ after many tests. The choice of this value can be adjusted accordingly. Other classifications are based on the fixed value region. Therefore, the region in (20) can be set:

(22) $$d_{k}\{\begin{array}{c} 0<max(d_{k})<0.5\\ 0.5<max(d_{k})<1\\ 1<max(d_{k})<10\\ 10<max(d_{k})<20\\ 20<max(d_{k})<30\\ 30<max(d_{k})<40\\ 40<max(d_{k})<50\\ \vdots \end{array}$$

After obtaining the residual range, the expectation of the squared residuals is defined as:

(23) $$\begin{array}{c} \begin{array}{cc} D_{k} & =E[d_{k}d_{k}^{T}]\\ \; & =TG{\hat{P}}_{k-1}^{y}G^{T}T^{T}+TQ_{k}^{y}T^{T}+R_{k}^{y}, \end{array} \end{array}$$

where G is a transition matrix of states defined as:

(24) $$G=\left[\begin{array}{cc} I_{M} & T_{s}I_{M}\\ 0_{M} & I_{M} \end{array}\right],$$

According to the characteristic of expectations, when the random variable X is in a uniform distribution, the expectation of the squared variable is:

(25) $$\begin{array}{c} \begin{array}{cc} E(X^{2}) & =\int_{a}^{b}\frac{X^{2}}{b-a}\mathrm{dX}\\ \; & =\frac{b^{2}+ab+b^{2}}{3}. \end{array} \end{array}$$

where *a* and *b* are the lower and upper bound of X, respectively.

Therefore, when the residual $d_{k}$ is in a uniform distribution, the expectation of the squared residual $D_{k}=diag\left(\left[\frac{b^{2}+ab+a^{2}}{3}\right]\right)$. In (20), the range of $d_{k}$ has been already classified, and thus, the corresponding $D_{k}$ is calculated by (25). Then, according to (23), the following equation is obtained:

(26) $$\begin{array}{c} TQ_{k}^{y}T^{T}+R_{k}^{y}=D_{k}-TG{\hat{P}}_{k-1}^{y}G^{T}T^{T}, \end{array}$$

where $TG{\hat{P}}_{k-1}^{y}G^{T}T^{T}$ can be calculated through the last-time estimation. $Q_{k}^{y}$ is generally not adjusted greatly, and the adjustment range is 0∼1. The parameter λ is defined as the adjustment factor of $Q_{k}^{y}$, which is defined as:

(27) $$\begin{array}{c} \lambda_{k,j}=\frac{N-j}{N}. \end{array}$$

where *N* is the number of range groups and *j* is the group to which it belongs.

When the residual $d_{k}$ is large, which means that the observed value contains a large noise component, the final estimate is biased toward the predicted value, so $Q_{k}^{y}$ needs to be reduced. Similarly, when the residual $d_{k}$ is small, the final estimate is biased towards the observed value, and $Q_{k}^{y}$ needs to be increased. The adjustment for $Q_{k}^{y}$ is shown below:

(28) $$\begin{array}{cc} \; & Q_{k}^{y}=\lambda_{k,j}\cdot Q_{0}^{y}\left(\lambda_{k,j}=\frac{N-j}{N}\right). \end{array}$$

**Figure 2:**
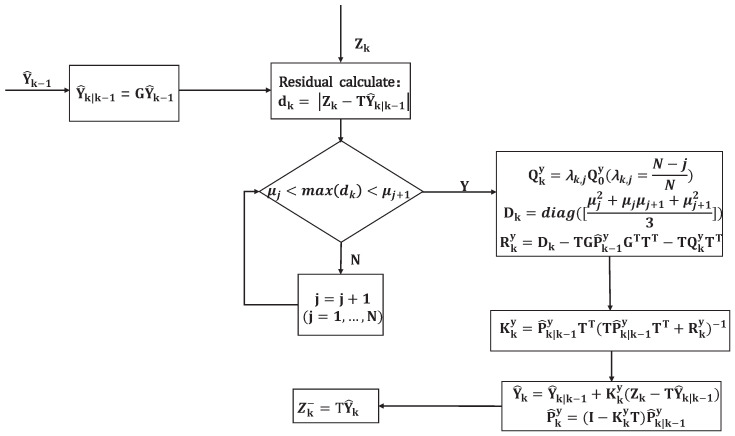
RCCA computational flowchart.

After the adjustment of $Q_{k}^{y}$ in (28), $R_{k}^{y}$ can be adjusted according to (26). Thus, residual classification and covariance adjustment are accomplished. One of the advantages of RCCA is that it does not require the detection and discrimination of LOS/NLOS measurements. The other advantage is that NLOS measurements can be retained, which is an indicator of the presence of NLOS interference for a long time. The flowchart of RCCA is shown in Figure 2.

## 4. Double Extended Kalman Filter Based on RCCA

In this section, residual classification and covariance adjustment (RCCA) is applied to the double extended Kalman filter, which is constructed by concatenating a KF and an EKF.

**Figure 3:**
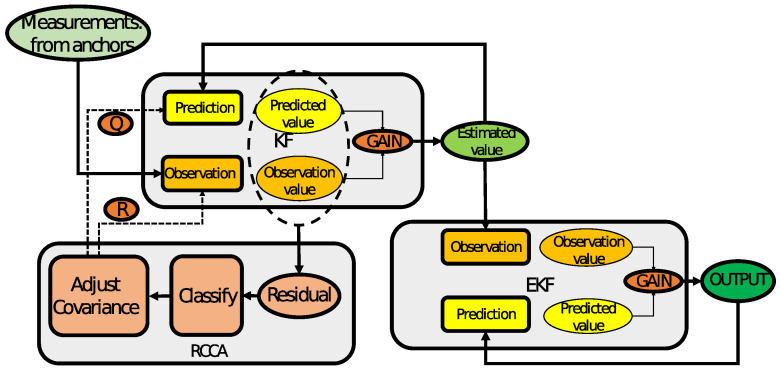
DEKF system framework diagram.

The main principle of RCCA is that the predicted value is obtained via a conventional filtering process through KF, and then, the residual between the predicted value and value (from the anchors) is obtained. The interval is classified according to the size of the residual, and the corresponding covariance matrix and observation noise covariance matrix are adjusted. Then, the results filtered by the KF are used as the input to the second EKF, which is used as the observation value and observation noise matrix of the EKF. The first-stage KF adjusts the covariance matrix through residual classification, to obtain more accurate observations for the second-stage EKF for further processing. The architecture of the overall system is shown in Figure 3.

First, for the pre-stage KF, the iteration calculation process of the KF is shown below:

1. Prediction:

(29) $$\begin{array}{c} {\hat{Y}}_{k|k-1}=G{\hat{Y}}_{k-1}, \end{array}$$

(30) $$\begin{array}{c} d_{k}=\left|Z_{k}-T{\hat{Y}}_{k|k-1}\right|, \end{array}$$

(31) $$\begin{array}{c} d_{k}\mathrm{classification}, \end{array}$$

(32) $$\begin{array}{c} Q_{k}^{y}=\lambda_{k,j}\cdot Q_{0}^{y}\left(\lambda_{k,j}=\frac{N-j}{N}\right), \end{array}$$

(33) $$\begin{array}{c} P_{k}^{y}=GP_{k-1}^{y}G^{T}+Q_{k}^{y}, \end{array}$$

2. RCCA:

(34) $$\begin{array}{c} R_{k}^{y}=D_{k}-TG{\hat{P}}_{k-1}^{y}G^{T}T^{T}-TQ_{k}^{y}T^{T}, \end{array}$$

3. Estimation:

(35) $$\begin{array}{c} K_{k}^{y}={\hat{P}}_{k|k-1}^{y}T^{T}{\left(T{\hat{P}}_{k|k-1}^{y}T^{T}+R_{k}^{y}\right)}^{-1}, \end{array}$$

(36) $$\begin{array}{c} {\hat{Y}}_{k}={\hat{Y}}_{k|k-1}+K_{k}^{y}d_{k}, \end{array}$$

(37) $$\begin{array}{c} {\hat{P}}_{k}^{y}=\left(I-K_{k}^{y}T\right){\hat{P}}_{k|k-1}^{y}. \end{array}$$

Then, for the latter EKF, the iteration process of the EKF is shown below:

1. Prediction

(38) $$\begin{array}{c} {\hat{X}}_{k|k-1}=A{\hat{X}}_{k-1}, \end{array}$$

(39) $$\begin{array}{c} {\hat{P}}_{k|k-1}^{x}=A{\hat{P}}_{k-1}^{x}A^{T}+Q_{k}^{x}, \end{array}$$

**Figure 4:**
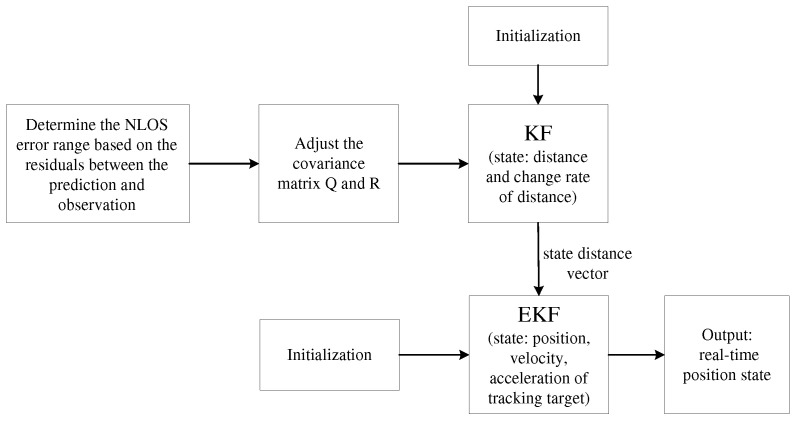
DEKF algorithm computational flowchart.

2. Estimation

(40) $$\begin{array}{c} R_{k}^{x}=T{\hat{P}}_{k}^{y}T^{T}, \end{array}$$

(41) $$\begin{array}{c} K_{k}^{x}={\hat{P}}_{k|k-1}^{x}H_{k}^{T}{\left(H_{k}{\hat{P}}_{k|k-1}^{x}H_{k}^{T}+R_{k}^{x}\right)}^{-1}, \end{array}$$

(42) $$\begin{array}{c} Z_{k}^{-}=T{\hat{Y}}_{k}, \end{array}$$

(43) $$\begin{array}{c} {\hat{X}}_{k}={\hat{X}}_{k|k-1}+K_{k}^{x}\left(Z_{k}^{-}-h\left({\hat{X}}_{k|k-1}\right)\right), \end{array}$$

(44) $$\begin{array}{c} {\hat{P}}_{k}^{x}=\left(I-K_{k}^{x}H_{k}\right){\hat{P}}_{k|k-1}^{x}. \end{array}$$

where the measurements $Z_{k}^{-}$ and the measured covariance matrix $R_{k}^{x}$ in the EKF system are based on the KF, and are calculated in (42) and (40). $Z_{k}^{-}$ is not the measurement from the anchors; instead, it is the estimation from the KF. The estimation error variance of the KF is selected as the corresponding EKF measurement covariance matrix $R_{k}^{x}$. The design uses more accurate distance values as the observations of the EKF, and the estimated state error variance decreases with iterations, which is in line with the trend of decreasing measurement error covariance. The double extended Kalman filter based on RCCA is summarized in Algorithm 1, and the flowchart is shown in Figure 4.
**Algorithm 1** Double-layer extended-Kalman filter algorithm.**Input:****KF:** The initial state (distance, rate of change for distance) of target $Y_{0}$ and the corresponding estimation error variance matrix $P_{0}^{y}$, the initial covariance $Q_{0}^{y}$ for Kalman filter process noise ensured by experiments, the measurements $Z_{k}$ of all distances between *M* stationary anchors and target.**EKF:** The initial state (position, velocity, acceleration) of the target $X_{0}$ and the corresponding estimation error variance matrix $P_{0}^{x}$, the initial covariance $Q_{0}^{x}$ for Extended Kalman filter process noise.The pre-set threshold $\mu_{1}$ for the first group classification in (20a).**For** k=1:times**First Kalman filter with RCCA:**- Calculate the prediction state ${\hat{Y}}_{k|k-1}$- Calculate the residual of prediction and measurements $d_{k}$ using (18)- Judge the range of $d_{k}$ using (22)- Adjust the $Q_{k}^{y}$ using (28)- Calculate the prediction variance ${\hat{P}}_{k|k-1}^{y}$ using (33)- Calculate the $R_{k}^{y}$ using (26)- Bring the adjusted $Q_{k}^{y}$ and $R_{k}^{y}$ into (35) to obtain Kalman gain $K_{k}^{y}$ of first KF- Output the distance state $T{\hat{Y}}_{k}$ and the estimation error variance $T{\hat{P}}_{k}^{y}T^{T}$.**Second Extended Kalman filter:**- Calculate the prediction state and covariance ${\hat{X}}_{k|k-1}$ and ${\hat{P}}_{k|k-1}^{x}$- Obtain the first KF’s output distance state as observation of second EKF $Z_{k}^{-}=T{\hat{Y}}_{k}$, and the estimation error variance as measurement covariance $R_{k}^{x}=T{\hat{P}}_{k}^{y}T^{T}$- Calculate the Kalman gain $K_{k}^{x}$ of second EKF- Update the state ${\hat{X}}_{k}$ and output- Update the estimation error variance ${\hat{P}}_{k}^{x}$ and output.**End For****Output:** ${\hat{X}}_{k}$, ${\hat{P}}_{k}^{x}$

## 5. Simulation Results and Experimental Verification

### 5.1. Simulation Environments and Settings

The simulation was carried out with MATLAB R2021b on a PC. The EKF, IMED-KF [19], M-REKF [30], and proposed DEKF algorithms were tested and compared under the CV and CA models. There are five stationary UWB anchors located at $\left[x_{1}^{U},y_{1}^{U},z_{1}^{U}\right]=\left[2\;m,7\;m,1\;m\right]$, $\left[x_{2}^{U},y_{2}^{U},z_{2}^{U}\right]=\left[12\;m,7\;m,2\;m\right],\left[x_{3}^{U},y_{3}^{U},z_{3}^{U}\right]=\left[7\;m,12\;m,3\;m\right],\left[x_{4}^{U},y_{4}^{U},z_{4}^{U}\right]=\left[7\;m,2\;m,5\;m\right]$, and $\left[x_{5}^{U},y_{5}^{U},z_{5}^{U}\right]=\left[7\;m,7\;m,7\;m\right]$. The simulation was performed with 100 Monte Carlo experiments with a time step of 0.01 s. The duration of each experiment was 10 s. The measurement noise was set to have a Gaussian distribution with $\left(\mu_{m},\sigma_{m}\right)=\left(0\;m,0.1\;m\right)$, and the threshold of the first interval was defined as $\mu_{1,i}=0.5,$ i=1,…,M, where M=5 is the number of anchors. The NLOS noise was modeled with a uniform distribution. The initial state of the second EKF was $X_{0}=[2\;m,2\;m,2\;m,0.4\;m/s,0.4\;m/s,0.4\;m/s,0.02\;m/s,0.02\;m/s,$${0.02\;m/s]}^{T}$, and the initial state error variance $P_{0}^{x}$ for the EKF was diag([0.1,0.1,0.1,0.01,0.01,0.01,0.005,0.005,0.005]). The distances in the initial state of the first KF were calculated by the initial target position and the anchor positions with $h_{i}\left(X_{k}\right)=\sqrt{{\left(x_{k}-x_{i}^{U}\right)}^{2}+{\left(y_{k}-y_{i}^{U}\right)}^{2}+{\left(z_{k}-z_{i}^{U}\right)}^{2}}$, and the variation rate of the distances $v_{d,0}$ was set to be 0.1m/s. The initial state error variance for the KF was defined as $P_{0}^{y}=diag\left(\left[0.1,\cdots ,0.1,0.01,\cdots ,0.01\right]\right)$.

**Figure 5:**
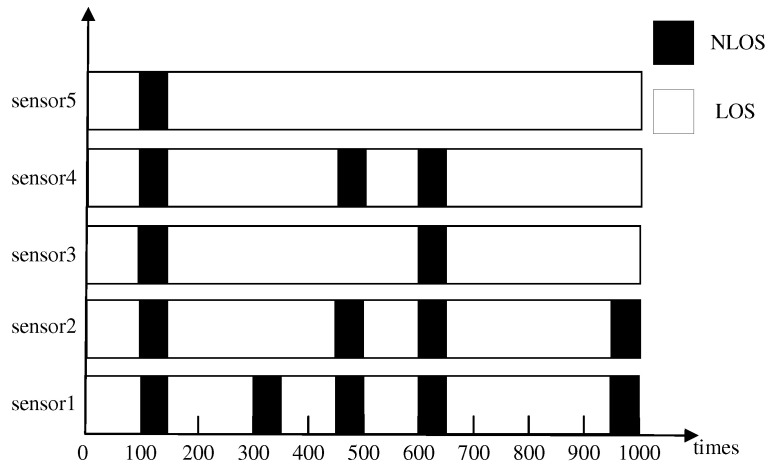
NLOS model distribution.

As mentioned in Section 2.2, uniformly distributed noise was chosen as the NLOS noise model. The NLOS noise model is described in Figure 5, where the black blocks represent the occupied time of the NLOS noise with U(0,10m) uniformly distributed noise, and the white blocks represent the occupied time in the LOS environment. Both the NLOS and LOS environments also have Gaussian measurement noise with $\left(\mu_{m},\sigma_{m}\right)=\left(0\;m,0.1\;m\right)$. The LOS/NLOS noise distribution for the five anchors in 1000 iteration times is shown in Figure 5.

### 5.2. Performance Metrics

The root mean squared error (RMSE) [39] is used to evaluate the accuracy of positioning, and it is defined as:

(45) $$RMSE_{k}=\sqrt{\frac{1}{L}\sum_{l=1}^{L}{\left(X_{l,k}-{\hat{X}}_{l,k}\right)}^{T}\left(X_{l,k}-{\hat{X}}_{l,k}\right)}.$$

where *L* is the number of Monte Carlo trials.

In addition, the Cramer–Rao lower bound (CRLB) [40,41,42] is also used as a metric for algorithm performance. Here, the Posterior CRLB (PCRLB) is used as the metric for $X_{k}$ estimation; it is defined as:

(46) $$E\left[\left(X_{k}-{\hat{X}}_{k}\right){\left(X_{k}-{\hat{X}}_{k}\right)}^{T}\right]\geq J_{k}^{-1}={\mathrm{PCRLB}}_{k},$$

where $J_{k}$ is the Fisher information matrix:

(47) $$J_{k}={\left[AJ_{k-1}^{-1}A^{T}+Q_{k-1}\right]}^{-1}+H_{k}^{T}R_{k}^{-1}H_{k}.$$

The cumulative distribution function (CDF), which is defined in (21), is also taken into account in the evaluation of target tracking accuracy.

### 5.3. Simulation Results

**Figure 6:**
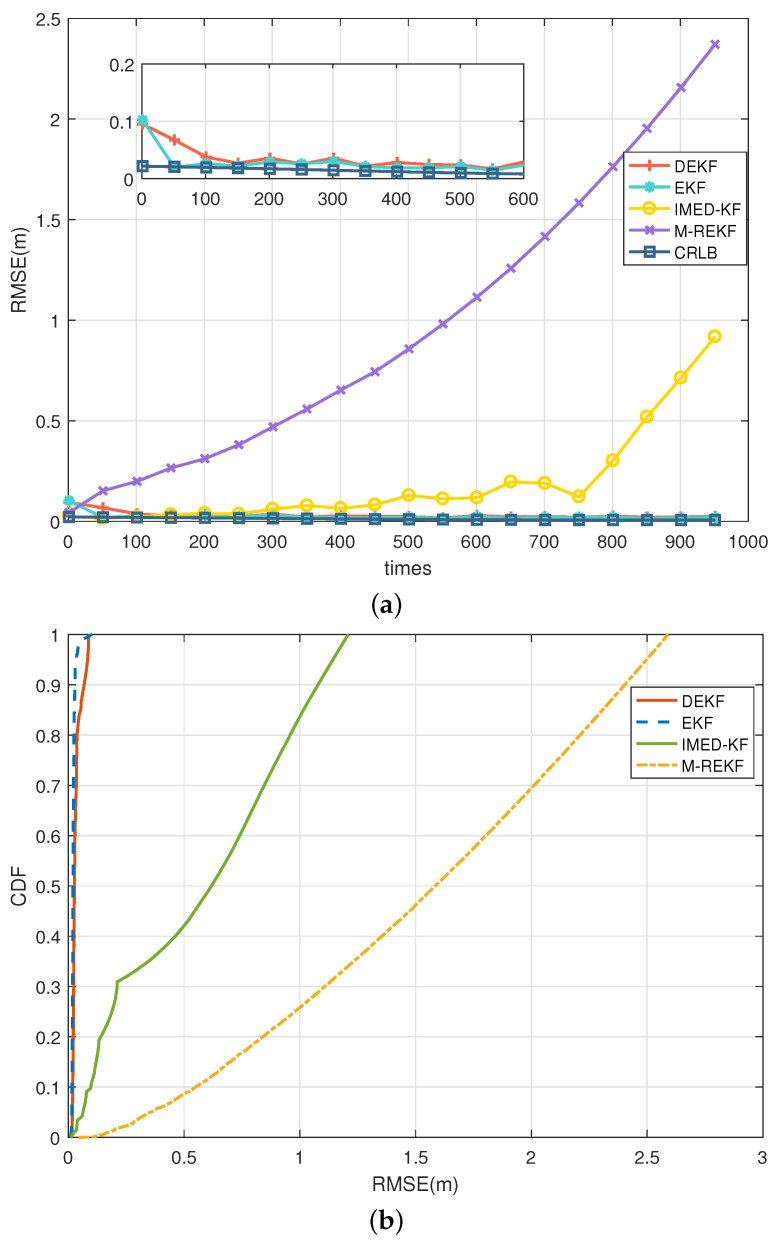
CA model: (**a**) RMSE comparison in LOS environment; (**b**) CDF comparison in LOS environment.

For generality, the CA model was used in the simulations, as shown in Figure 6, in which the RMSE and CDF of different filtering results are presented in the environment where the noise of all five anchors contains LOS only. In the LOS environment, EKF and the proposed DEKF can have a steady-ranging accuracy within 10 cm, while IMED-KF and M-REKF diverge during iteration. Using the two-state Markov chain described in Section 2.2 as the NLOS/LOS environment simulation model, four different scenarios, S1–S4, were set, and the NLOS noise distribution of the five anchors in different scenarios is as follows:

S1: ε=[0.1,0,0,0,0],
S2: ε=[0,0.25,0,0.25,0],
S3: ε=[0,0.25,0.1,0.75,0],
S4: ε=[0.25,0.25,0.25,0.25,0.25].

**Figure 7:**
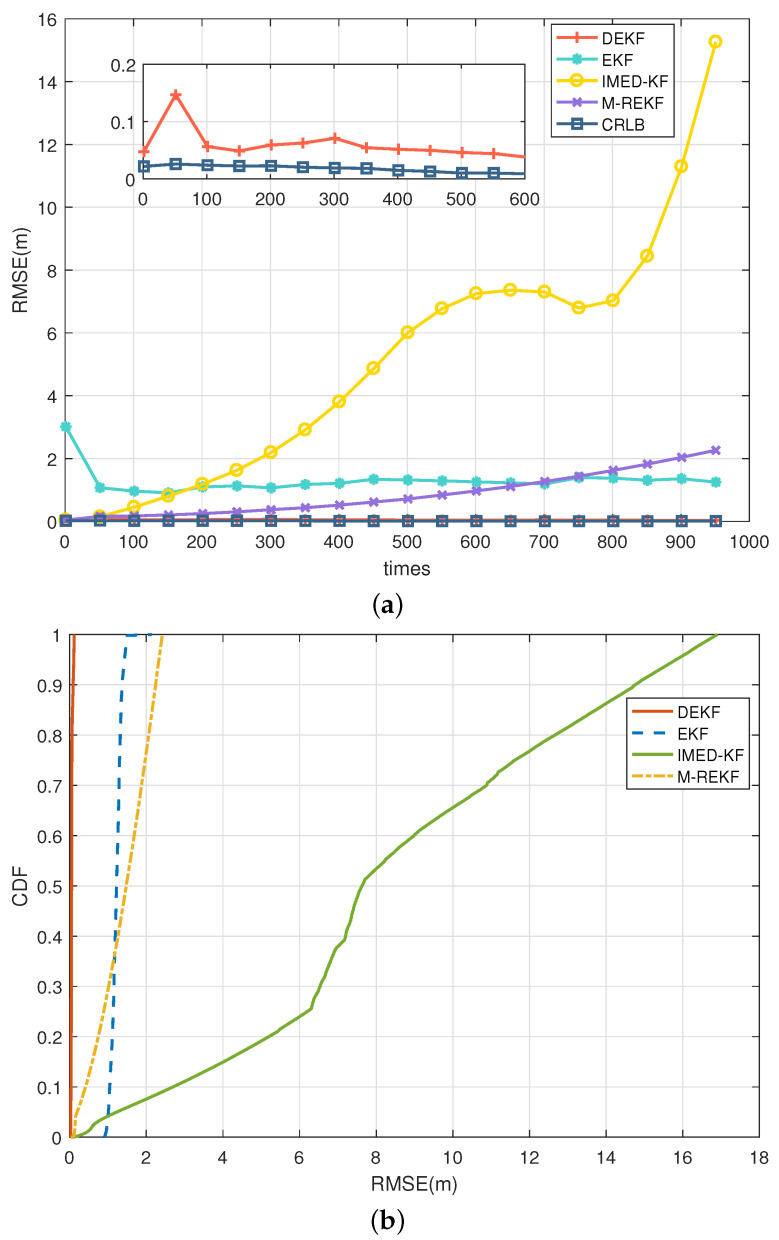
CA model: (**a**) RMSE comparison in LOS/NLOS environment S4; (**b**) CDF comparison in LOS/NLOS environment S4.

In scenario S4, the results in the CA model present different outcomes, as shown in Figure 7. In the CA model, the RMSE of IMED-KF grows fast during iteration, and M-REKF also tends to increase with a smaller slope. On the contrary, the proposed DEKF is stable within 5 cm, and EKF is stable with a meter-level RMSE. The simulation results illustrate that IMED-KF and M-REKF in the CA motion model diverge much more easily, leading to an inaccurate result. However, the proposed DEKF is stable and highly accurate in the CA model. The tracking RMSE results in the LOS and S1–S4 environments for the CA model are illustrated in Table 2. It can be seen that DEKF can remain stable and have a much more accurate range when the environment changes, while EKF, IMED-KF, and M-REKF are far from accurate.

### 5.4. Experimental Verification of the Algorithm

**Figure 8:**
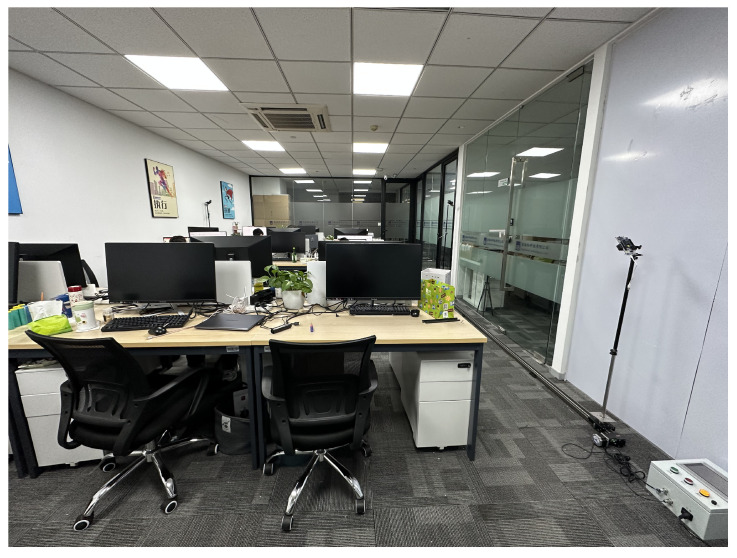
The environment of the test office.

In order to test the performance of the proposed algorithm in a complex indoor environment, an experimental environment was built in our office using a UWB module, including four anchors and one target. The office area is about $46\;m^{2}\;\left(4.76\times 9.63\;m\right)$, and there were many people and some equipment in the office when these experiments were carried out, as shown in Figure 8.

**Figure 9:**
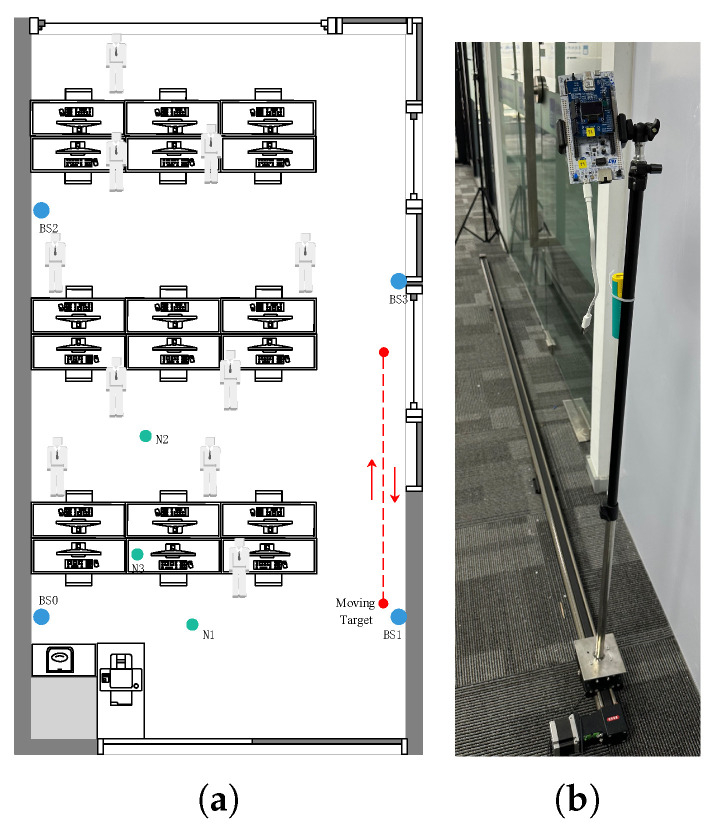
(**a**) Trajectory in indoor office environment. (**b**) Test track in indoor office environment.

The DEKF, EKF, and IMED-KF algorithms are used for performance comparison. As shown in Figure 9a, red lines and red dots represent the position and trajectory of the target, and green dots represent stationary points. Blue points represent the anchors, and their coordinates are $BS_{1}\left(0.20,1.68\right)\;m,BS_{2}\left(4.45,1.68\right)\;m,BS_{3}\left(0.20,7.15\right)\;m$, and $BS_{4}\left(4.45,6.18\right)\;m$. The anchor height was set to 1.7m to aid us in building the experimental environment in the lab, such as adding metal sheets to block the anchors for NLOS occurrence in tracking experiments. Figure 9b shows the test track in the indoor office environment.

The dashed line in Figure 9a shows the motion of the target. The target performs a 3m reciprocating motion. Its *x*-axis remains the same, while the *y*-axis is displaced up to 6m.

**Figure 10:**
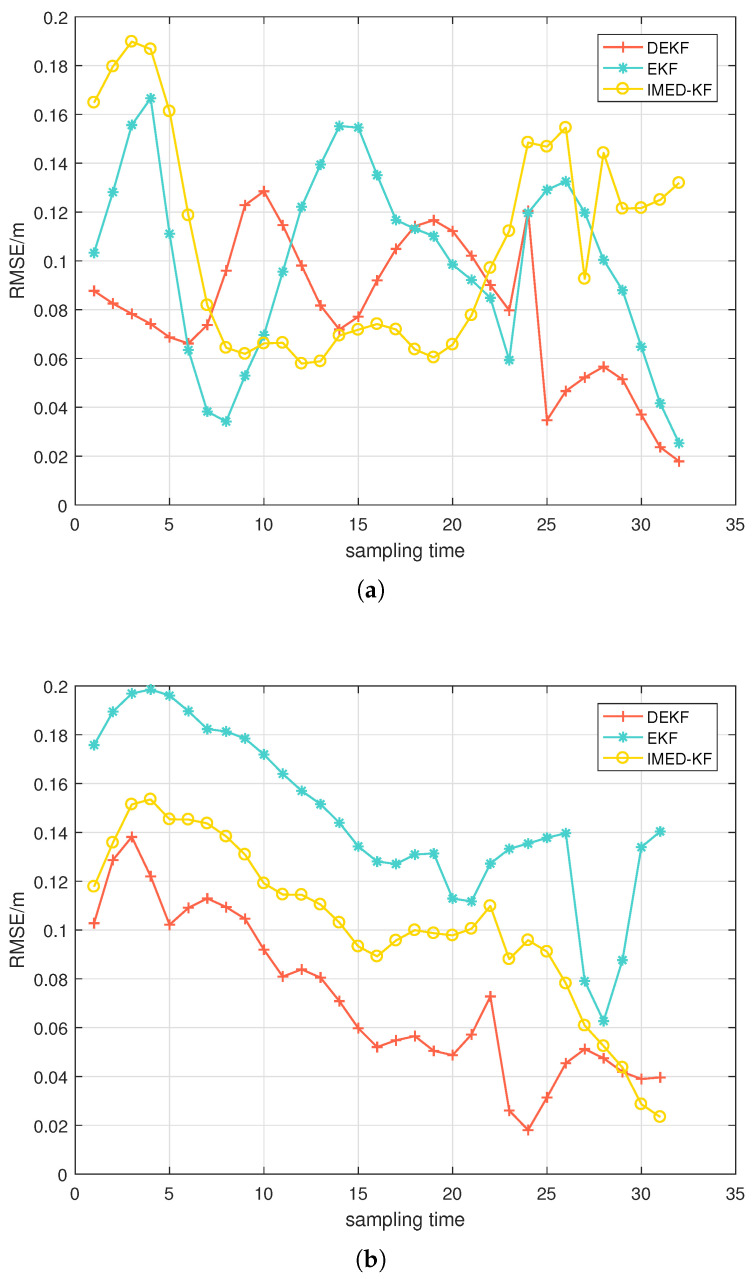
CV model: (**a**) RMSE in the LOS situation; (**b**) RMSE in four NLOS situations.

Figure 10 illustrates the RMSE values of several algorithms mentioned in the previous section for the LOS environment and the four NLOS cases. The RMSEs of these algorithms in the LOS environment are very close, under 10 cm, which means that the three algorithms are all accurate enough to achieve real-time tracking. In the four NLOS environment cases, the RMSEs of the three algorithms are relatively small, and the RMSE of DEKF is the smallest.

**Figure 11:**
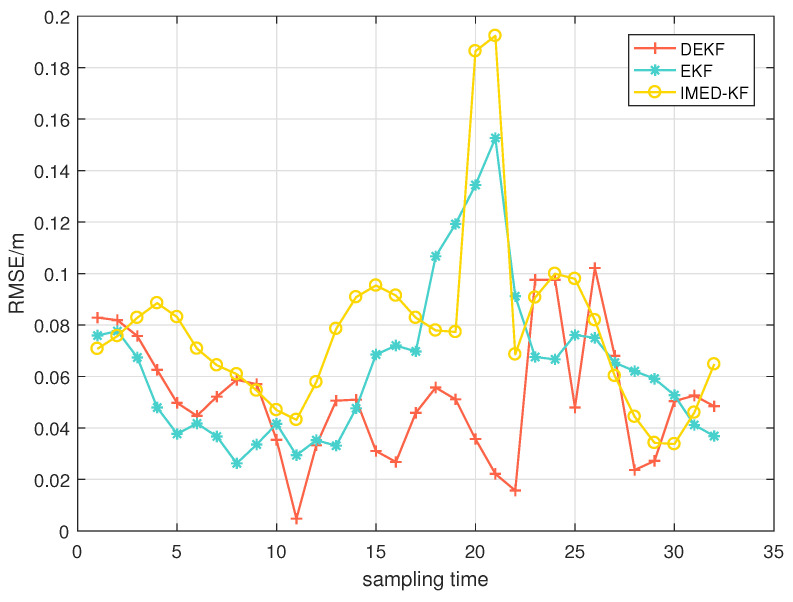
CV model: RMSE after change from 1 to NLOS to 2-NLOS.

Figure 11 shows the RMSEs of each algorithm when changing the number of anchors subject to NLOS interference from one to two. When the target returns from the other side of the track, an NLOS anchor is added for a duration of about half the experiment time. The RMSE of EKF and IMD-KF increases rapidly at half time, while DEKF remains stable when the number of NLOS anchors increases.

For the CA model, the results in the LOS and NLOS situations are shown in Table 3. Among the three algorithms, IMED-KF cannot obtain a stable and accurate result in this motion model, whereas the proposed DEKF and EKF can track the target in a relatively accurate way. In the LOS environment, DEKF and EKF can reach high accuracy with values close to each other. When the number of NLOS anchors increases, EKF tracks the target more and more inaccurately, especially in the 4-NLOS situation. However, the RMSE of DEKF can remain stable when the environment become severe, with an average RMSE from 17 cm to 26 cm.

## 6. Conclusions

This article proposes a double extended Kalman filter algorithm in order to fix the weakening effect on tracking accuracy when the measurements have NLOS and measuring noises. The preceding Kalman filter with a residual classification and covariance adjustment (RCCA) module was designed to smooth the distance input and can adjust the Kalman gain in time as the observations change. Then, the states of the preceding KF into the next EKF as the distance observations change. The experimental results demonstrate that the algorithm presented in this paper exhibits high accuracy under both CV and CA models. It can achieve centimeter-level accuracy in LOS/NLOS environments, specifically, an accuracy within 4 cm in LOS and within 10 cm in NLOS, and it converges at a faster speed than its counterparts according to the results of numerous simulations. Using the proposed algorithm, target tracking will be performed quite accurately when the LOS measurements are diminished near to zero. In addition, the accuracy will not decrease significantly when severe NLOS errors lasting a long time occur, as verified by the simulation and experimental results.

## Figures and Tables

**Table 1:** Scaling factor of NLOS noise.

$\varepsilon_{i}$	α	β
0.1	0.01	0.09
0.25	0.02	0.06
0.5	0.05	0.05
0.75	0.06	0.02

**Table 2:** CA model: positioning result comparison in LOS and S1–S4.

Noise Situation	Tracking Results: Average RMSE/m			
	DEKF	EKF	IMED-KF	M-REKF
LOS	0.022	0.017	0.181	2.227
S1	0.023	0.228	0.232	2.245
S2	0.027	0.926	3.385	2.327
S3	0.052	1.453	8.647	2.408
S4	0.054	1.868	11.934	2.467

**Table 3:** Positioning test result comparison in CA model.

NLOS Number	Tracking Results/m	
	DEKF	EKF
LOS	tracking	tracking
	${\mathrm{RMSE}}_{max}=0.33$	${\mathrm{RMSE}}_{max}=0.53$
	${\mathrm{RMSE}}_{avg}=0.17$	${\mathrm{RMSE}}_{avg}=0.18$
1-NLOS	tracking	tracking
	${\mathrm{RMSE}}_{max}=0.37$	${\mathrm{RMSE}}_{max}=0.69$
	${\mathrm{RMSE}}_{avg}=0.18$	${\mathrm{RMSE}}_{avg}=0.27$
2-NLOS	tracking	tracking
	${\mathrm{RMSE}}_{max}=0.39$	${\mathrm{RMSE}}_{max}=0.71$
	${\mathrm{RMSE}}_{avg}=0.18$	${\mathrm{RMSE}}_{avg}=0.34$
3-NLOS	tracking	tracking
	${\mathrm{RMSE}}_{max}=0.54$	${\mathrm{RMSE}}_{max}=0.89$
	${\mathrm{RMSE}}_{avg}=0.24$	${\mathrm{RMSE}}_{avg}=0.41$
4-NLOS	tracking	tracking
	${\mathrm{RMSE}}_{max}=0.61$	${\mathrm{RMSE}}_{max}=255.48$
	${\mathrm{RMSE}}_{avg}=0.26$	${\mathrm{RMSE}}_{avg}=53.56$

## Data Availability

Data are contained within the article.
